# A new hybrid Dawson-type molybdenum arsenate derivative: (H_2_bpy)_3_[As_2_Mo_18_O_62_] (bpy = 4,4′-bipyridine)

**DOI:** 10.1107/S1600536809050260

**Published:** 2009-11-28

**Authors:** Hai-hui Yu, Xiao Zhang, Li Kong, Ji-qing Xu

**Affiliations:** a College of Chemical Engineering, North-East Dianli University, Jilin 132012, People’s Republic of China; b The Academy of Fundamental and Interdisciplinary Sciences, Harbin Institute of Technology, Rm 403, Bldg 2G, 2 Yikuang St, Nangang District, Harbin 150080, People’s Republic of China; c Jilin Institute of Chemical Technology, Jilin 132012, People’s Republic of China; d College of Chemistry and State Key Laboratory of Inorganic Synthesis and Preparative Chemistry, Jilin University, Changchun 130023, People’s Republic of China

## Abstract

The title compound, tris­(4,4′-bipyridinium) diarsenoocta­deca­molybdate(VI), (C_10_H_10_N_2_)_3_[As_2_Mo_18_O_62_], featuring protonated bipyridine mol­ecules and a classical Dawson-type polyoxo-anion, has been synthesized under hydro­thermal conditions. The polyoxoanions are linked together *via* the bipyridyl cations, acting as hydrogen-bond donors, generating a two-dimensional supra­molecular network. The asymmetric unit contains 1.5 4,4′-bipyridinium (H_2_bpy) units, with an inversion centre in the central bond of the second H_2_bpy unit. The site symmetry of the anion is 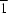.

## Related literature

For the use of polyoxometalates in the construction of functional materials, see: Haushalter *et al.* (1989 ▶); Pope & Müller (1991 ▶). For *A*/Mo/P/O compounds where *A* is an organic or inorganic cation, see: Rao *et al.* (2001 ▶); Cheetham *et al.* (1999 ▶); Thomas & Raja (2001 ▶); Xiao *et al.* (1999 ▶). For Dawson-type polyoxometalates, see: Wang *et al.* (2004 ▶).
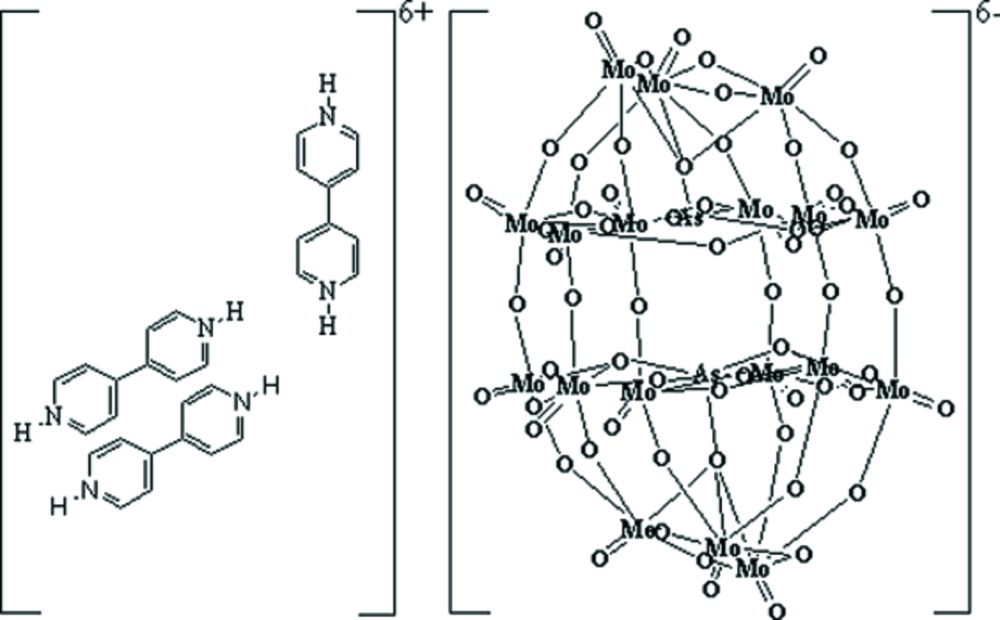


## Experimental

### 

#### Crystal data

(C_10_H_10_N_2_)_3_[As_2_Mo_18_O_62_]
*M* *_r_* = 3343.36
Triclinic, 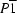

*a* = 11.2671 (17) Å
*b* = 12.1365 (19) Å
*c* = 13.871 (2) Å
α = 108.023 (2)°
β = 94.243 (2)°
γ = 107.166 (2)°
*V* = 1694.6 (4) Å^3^
*Z* = 1
Mo *K*α radiation
μ = 4.30 mm^−1^
*T* = 293 K
0.29 × 0.22 × 0.20 mm

#### Data collection

Rigaku R-AXIS RAPID diffractometer
Absorption correction: multi-scan (*ABSCOR*; Higashi, 1995 ▶) *T*_min_ = 0.504, *T*_max_ = 0.625
14379 measured reflections
6545 independent reflections
4986 reflections with *I* > 2σ(*I*)
*R*_int_ = 0.042

#### Refinement

*R*[*F* ^2^ > 2σ(*F* ^2^)] = 0.047
*wR*(*F* ^2^) = 0.128
*S* = 1.06
6545 reflections
532 parameters
H-atom parameters constrained
Δρ_max_ = 3.42 e Å^−3^
Δρ_min_ = −1.79 e Å^−3^

### 

Data collection: *RAPID-AUTO* (Rigaku, 1998 ▶); cell refinement: *RAPID-AUTO*; data reduction: *RAPID-AUTO*; program(s) used to solve structure: *SHELXS97* (Sheldrick, 2008 ▶); program(s) used to refine structure: *SHELXTL* (Sheldrick, 2008 ▶); molecular graphics: *ORTEP-3* (Burnett & Johnson, 1996 ▶); software used to prepare material for publication: *SHELXL97* (Sheldrick, 2008 ▶).

## Supplementary Material

Crystal structure: contains datablocks I, global. DOI: 10.1107/S1600536809050260/fi2091sup1.cif

Structure factors: contains datablocks I. DOI: 10.1107/S1600536809050260/fi2091Isup2.hkl

Additional supplementary materials:  crystallographic information; 3D view; checkCIF report

## Figures and Tables

**Table 1:** Hydrogen-bond geometry (Å, °)

*D*—H⋯*A*	*D*—H	H⋯*A*	*D*⋯*A*	*D*—H⋯*A*
N1—H1*A*⋯O14	0.86	1.99	2.851 (10)	174
N2—H2*B*⋯O29^i^	0.86	2.32	2.805 (12)	116
N3—H3*B*⋯O10^ii^	0.86	2.56	3.291 (9)	143
N3—H3*B*⋯O7^ii^	0.86	2.60	3.336 (10)	145

Symmetry codes: (i) 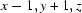; (ii) 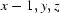.

**Fig. 1.**
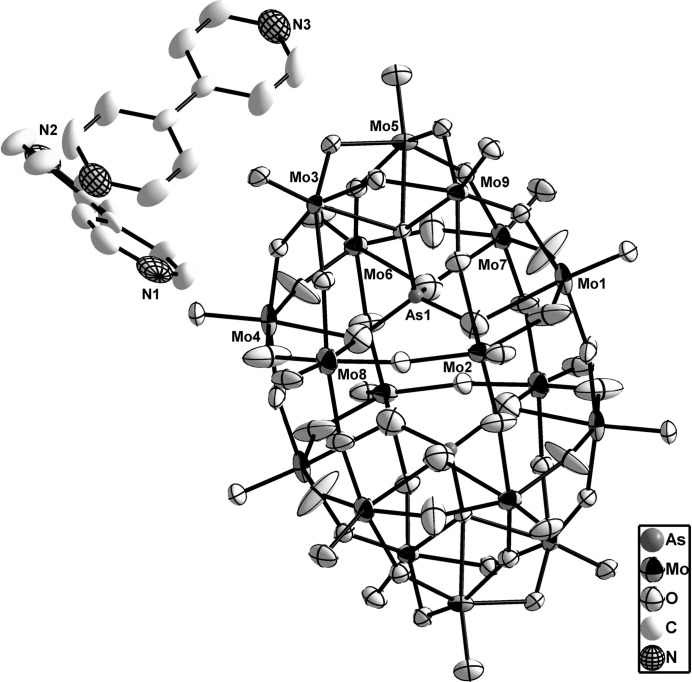
A view ofthe molecule of (I) with displacement ellipsoids drawn at the 30% probability level. H atoms have been omitted. Second half of the anion generated by (i) 2–x, 1–y, 1–z. Second half of the second, centrosymmetric cation generated by (ii) 1–x, 1–y, –z.

**Fig. 2.**
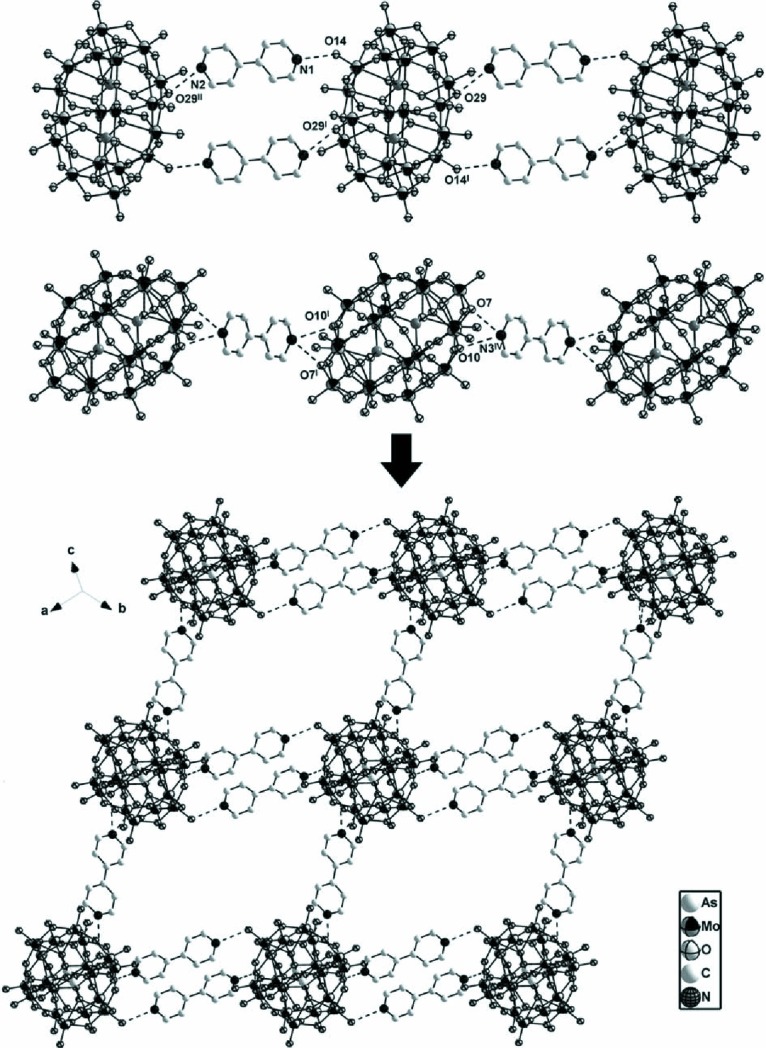
A polyhedral representation of the supramolecular layerconstructed by "double-bridge" chain A and chain B. Symmetry codes (i) 2 – x, 1 – y, 1 – z; (ii) x – 1, y + 1, z; (iv) 1 – x, 1 – y, 1 – z.

## supplementary crystallographic information

### Comment

POMs (polyoxometalates), as a class of metal oxide clusters, possess an enormous structural variety and interesting electronic properties. They have therefore been extensively employed in the construction of functional materials (Haushalter *et al.*, 1989; Pope & Müller, 1991). The A/Mo/P/O system as an important part of this family of compounds has been synthesized and structurally characterized, where A is an organic or inorganic cation. In contrast to the rich structural chemistry of molybdenum phosphates, the Mo/As/O system remains relatively scarcely developed.(Rao *et al.*, 2001; Cheetham *et al.*, 1999; Xiao *et al.*, 1999; Thomas & Raja 2001.) On the basis of these facts, we have hydrothermally synthesized and characterised a new arsenate compound (H_2_bpy)_3_[As_2_Mo_18_O_62_], which is described here.

The structure of compound consists of a discrete polyoxoanion [As_2_Mo_18_O_62_]^6-^ and three bipyridine molecules (Fig 1). The centrosymmetric polyoxoanion is classical α-Dawson isomer [α-As_2_Mo_18_O_62_]^6-^. The parent anion [α-As_2_Mo_18_O_62_]^6-^consisting of two central AsO_4_ tetrahedra which are surrounded by six vertex-sharing Mo_3_O_13_ trimers, also may be described as two [α-AsMo_9_O_31_]^3-^ units, generated from the well known [α-AsMo_12_O_40_]^3-^ by removal of a set of three corner-sharing MoO_6_ octahedra and fused into a clusterof virtual *D_3h_* symmetry. In the Dawson type POM, there are two structurally distinct types of Mo atoms: six 'cap' atoms on vertical mirror-planes and grouped in two sets of three, and twelve equatorial Mo atoms are grouped in two sets of six, but do not lie on mirror-planes (Wang *et al.*, 2004).

The unusual feature of the title compound is that it exhibits a 2-D supramolecular layer-like structure formed by the discrete Dawson-type anions and 4,4'-bpy molecules *via* multi-point N—H···O hydrogen-bonding interactions (Fig. 2). As a fundamental building unit, each of the polyoxoanion [As_2_Mo_18_O_62_]^6-^ acting as a hexa-dentate ligand furnishes four two-bridging and two terminal oxygen atoms to connect to six adjacent 4,4'-bipy molecules *via* N—H···O hydrogen-bonding interactions. In particular, oxygen atoms (O14, O26^i^, O29 and O12^i^; symmetry code: (i) *x* - 1, 1 + *y*, *z)* connect adjacent four 4,4'-bipy molecules to form a 1-D "double-bridge" chain (chain A), respectively. The oxygen atoms (O7, O10, O7^i ^and O10^i^) link two 4,4'-bpy molecules to construct an undulated chain (chain B). The chains A and B further link each other to forma novel 2-D layer-like structure with 1-D rhombic channel.

### Experimental

The title compound was hydrothermally synthesized under autogenous pressure. A mixture of FeCl_3_.6H_2_O (0.41 g, 2.5 mmol), As_2_O_3_(0.5 g, 2.5 mmol), MoO_3_.2H_2_O(0.9 g, 5 mmol), 4,4'-bipy.2H_2_O (0.32 g, 1.7 mmol) and 18 ml water was stirred for 30 min in air; it was adjusted to pH=5–6 with 2*M* KOH, and was heated in a 25 ml stainless steel reactor with a Teflon-liner at180°C for 8 days, and then cooled to room temperature. The resulting product consisting of brown block-shaped crystals was isolated by filtration, washed with distilled water, and dried at ambient temperature (75% yield based on Mo). Elemental analysis for 1: Anal. Calcd: C, 10.77; H, 0.8970; N, 2.52; found: C, 10.96; H, 0.923; N, 2.50. F T-IRdate: (KBr pellet, ν/cm^-1^): 3096(w),3069(w), 1621(*m*),1596(*m*), 1546(*m*), 1488(*m*), 1418(w), 1349(w), 1204(w), 950 (*m*), 930 (*m*), 888(*s*), 842 (*s*), 776(*s*), 724 (*s*).

### Refinement

All H atoms were placed at calculated positions (H—C = 0.93 Å), with *U*_iso_(H) = 1.2 *U*_eq_(C) and (H—N = 0.86 Å), with *U*_iso_(H) =1.2*U*_eq_(N).

### Crystal data

**Table:**

(C_10_H_10_N_2_)_3_[As_2_Mo_18_O_62_]	*Z* = 1
*M**_r_* = 3343.36	*F*(000) = 1569
Triclinic, *P*1	*D*_x_ = 3.276 Mg m^−^^3^
Hall symbol: -P 1	Mo *K*α radiation, λ = 0.71073 Å
*a* = 11.2671 (17) Å	Cell parameters from 4839 reflections
*b* = 12.1365 (19) Å	θ = 3.2–26.6°
*c* = 13.871 (2) Å	µ = 4.30 mm^−^^1^
α = 108.023 (2)°	*T* = 293 K
β = 94.243 (2)°	Block, brown
γ = 107.166 (2)°	0.29 × 0.22 × 0.20 mm
*V* = 1694.6 (4) Å^3^	

### Data collection

**Table:**

Rigaku R-AXIS RAPID diffractometer	6545 independent reflections
Radiation source: fine-focus sealed tube	4986 reflections with *I* > 2σ(*I*)
graphite	*R*_int_ = 0.042
Detector resolution: 10 pixels mm^-1^	θ_max_ = 26.1°, θ_min_ = 2.2°
ω scans	*h* = −13→13
Absorption correction: multi-scan (*ABSCOR*; Higashi, 1995)	*k* = −15→14
*T*_min_ = 0.504, *T*_max_ = 0.625	*l* = −17→17
14379 measured reflections	

### Refinement

**Table:**

Refinement on *F*^2^	Primary atom site location: structure-invariant direct methods
Least-squares matrix: full	Secondary atom site location: difference Fourier map
*R*[*F*^2^ > 2σ(*F*^2^)] = 0.047	Hydrogen site location: inferred from neighbouring sites
*wR*(*F*^2^) = 0.128	H-atom parameters constrained
*S* = 1.06	*w* = 1/[σ^2^(*F*_o_^2^) + (0.0669*P*)^2^] where *P* = (*F*_o_^2^ + 2*F*_c_^2^)/3
6545 reflections	(Δ/σ)_max_ = 0.001
532 parameters	Δρ_max_ = 3.42 e Å^−^^3^
0 restraints	Δρ_min_ = −1.79 e Å^−^^3^

### Special details

**Table:**

Experimental. (See detailed section in the paper)
Geometry. All e.s.d.'s (except the e.s.d. in the dihedral angle between two l.s. planes) are estimated using the full covariance matrix. The cell e.s.d.'s are taken into account individually in the estimation of e.s.d.'s in distances, angles and torsion angles; correlations between e.s.d.'s in cell parameters are only used when they are defined by crystal symmetry. An approximate (isotropic) treatment of cell e.s.d.'s is used for estimating e.s.d.'s involving l.s. planes.
Refinement. Refinement of *F*^2^ against ALL reflections. The weighted *R*-factor *wR* and goodness of fit *S* are based on *F*^2^, conventional *R*-factors *R* are based on *F*, with *F* set to zero for negative *F*^2^. The threshold expression of *F*^2^ > σ(*F*^2^) is used only for calculating *R*-factors(gt) *etc*. and is not relevant to the choice of reflections for refinement. *R*-factors based on *F*^2^ are statistically about twice as large as those based on *F*, and *R*- factors based on ALL data will be even larger.

### Fractional atomic coordinates and isotropic or equivalent isotropic displacement parameters (Å^2^)

**Table:**

	*x*	*y*	*z*	*U*_iso_*/*U*_eq_	
As1	0.92601 (6)	0.39285 (6)	0.35346 (5)	0.01514 (17)	
Mo1	0.92713 (7)	0.13757 (7)	0.42221 (6)	0.0338 (2)	
Mo2	1.20090 (6)	0.30431 (6)	0.35916 (5)	0.02772 (18)	
Mo3	0.89323 (7)	0.36087 (6)	0.08380 (5)	0.02746 (18)	
Mo4	0.93667 (7)	0.65754 (7)	0.29565 (6)	0.0334 (2)	
Mo5	0.64553 (6)	0.19759 (6)	0.16132 (5)	0.02573 (17)	
Mo6	0.65550 (6)	0.48482 (6)	0.36298 (5)	0.02779 (18)	
Mo7	0.66101 (7)	0.24692 (7)	0.44528 (6)	0.0339 (2)	
Mo8	1.18997 (7)	0.55435 (7)	0.27224 (6)	0.0338 (2)	
Mo9	0.91060 (6)	0.11763 (6)	0.14911 (5)	0.02525 (17)	
O1	0.8708 (5)	0.0839 (5)	0.2713 (4)	0.0242 (11)	
O2	0.8653 (4)	0.3000 (4)	0.2292 (3)	0.0188 (10)	
O3	0.8780 (5)	0.5139 (4)	0.1693 (4)	0.0253 (12)	
O4	0.6376 (5)	0.3687 (5)	0.2392 (4)	0.0253 (12)	
O5	1.0703 (5)	0.4379 (4)	0.1562 (4)	0.0251 (12)	
O6	0.6331 (5)	0.1776 (5)	0.2873 (4)	0.0254 (11)	
O7	0.9234 (5)	0.2055 (4)	0.0511 (4)	0.0245 (11)	
O8	1.2798 (5)	0.3941 (5)	0.4871 (4)	0.0421 (16)	
O9	1.3038 (5)	0.2367 (5)	0.3112 (4)	0.0348 (14)	
O10	1.0727 (5)	0.1992 (5)	0.2114 (4)	0.0292 (12)	
O11	0.7206 (5)	0.0832 (5)	0.1100 (4)	0.0262 (12)	
O12	0.7180 (5)	0.2819 (5)	0.0661 (4)	0.0262 (12)	
O13	0.7181 (5)	0.3320 (6)	0.5857 (4)	0.0394 (15)	
O14	0.9134 (6)	0.7594 (5)	0.2412 (4)	0.0340 (14)	
O15	0.9939 (6)	0.2245 (5)	0.5659 (4)	0.0352 (14)	
O16	0.5297 (5)	0.1427 (5)	0.4500 (4)	0.0380 (15)	
O17	0.4954 (5)	0.1292 (5)	0.0943 (4)	0.0364 (14)	
O18	0.9085 (5)	−0.0231 (5)	0.0725 (4)	0.0310 (13)	
O19	0.9045 (5)	0.3812 (5)	−0.0306 (4)	0.0335 (13)	
O20	0.8980 (6)	−0.0045 (5)	0.4235 (4)	0.0395 (15)	
O21	1.3061 (5)	0.6043 (5)	0.2118 (4)	0.0394 (15)	
O22	0.5319 (5)	0.5286 (6)	0.3467 (4)	0.0428 (16)	
O23	1.0144 (6)	0.3297 (6)	0.4072 (5)	0.0480 (16)	
O24	0.8062 (6)	0.4072 (6)	0.4183 (5)	0.0489 (17)	
O25	0.7783 (7)	0.1653 (9)	0.4418 (5)	0.079 (3)	
O26	1.0145 (6)	0.5316 (6)	0.3549 (5)	0.0506 (17)	
O27	1.2249 (8)	0.4323 (5)	0.3085 (6)	0.077 (3)	
O28	1.1036 (7)	0.6686 (7)	0.2723 (6)	0.073 (3)	
O29	1.0918 (6)	0.1676 (8)	0.3915 (5)	0.071 (3)	
O30	0.5883 (10)	0.3638 (6)	0.4230 (5)	0.078 (3)	
O31	0.7859 (7)	0.6060 (6)	0.3357 (6)	0.073 (3)	
C1	0.6467 (10)	0.8466 (8)	0.1693 (8)	0.047 (2)	
H1B	0.6924	0.8474	0.1163	0.056*	
C2	0.6260 (9)	0.8143 (9)	0.3263 (8)	0.047 (2)	
H2A	0.6561	0.7914	0.3787	0.056*	
C3	0.5380 (9)	0.8722 (7)	0.1670 (7)	0.038 (2)	
H3A	0.5085	0.8904	0.1114	0.046*	
C4	0.5159 (9)	0.8432 (9)	0.3276 (7)	0.046 (2)	
H4A	0.4734	0.8434	0.3826	0.056*	
C5	0.1959 (12)	0.9608 (10)	0.3356 (10)	0.061 (3)	
H5A	0.1603	0.9753	0.3947	0.074*	
C6	0.2963 (11)	0.9183 (10)	0.1629 (7)	0.059 (3)	
H6A	0.3286	0.9036	0.1021	0.071*	
C7	0.1878 (11)	0.9533 (10)	0.1679 (8)	0.063 (3)	
H7A	0.1484	0.9632	0.1113	0.075*	
C8	0.3549 (8)	0.9057 (7)	0.2474 (7)	0.037 (2)	
C9	0.4693 (8)	0.8718 (7)	0.2464 (6)	0.034 (2)	
C10	0.3004 (10)	0.9289 (9)	0.3353 (8)	0.048 (2)	
H10A	0.3370	0.9222	0.3946	0.058*	
C11	0.4424 (8)	0.4594 (8)	0.0113 (6)	0.0334 (19)	
C12	0.4500 (10)	0.3954 (11)	0.0767 (9)	0.058 (3)	
H12A	0.5292	0.4027	0.1080	0.070*	
C13	0.3450 (11)	0.3218 (10)	0.0970 (9)	0.058 (3)	
H13A	0.3528	0.2779	0.1401	0.070*	
C14	0.2188 (8)	0.3792 (10)	−0.0083 (7)	0.045 (2)	
H14A	0.1391	0.3758	−0.0350	0.054*	
C15	0.3229 (9)	0.4474 (9)	−0.0299 (7)	0.043 (2)	
H15A	0.3139	0.4886	−0.0750	0.052*	
N1	0.6874 (8)	0.8199 (7)	0.2501 (7)	0.055 (2)	
H1A	0.7577	0.8056	0.2520	0.066*	
N2	0.1441 (8)	0.9716 (7)	0.2529 (8)	0.058 (2)	
H2B	0.0772	0.9919	0.2553	0.069*	
N3	0.2332 (8)	0.3133 (7)	0.0553 (6)	0.049 (2)	
H3B	0.1671	0.2660	0.0674	0.059*	

### Atomic displacement parameters (Å^2^)

**Table:**

	*U*^11^	*U*^22^	*U*^33^	*U*^12^	*U*^13^	*U*^23^
As1	0.0152 (4)	0.0145 (4)	0.0146 (4)	0.0032 (3)	0.0002 (3)	0.0058 (3)
Mo1	0.0392 (4)	0.0272 (4)	0.0504 (5)	0.0167 (3)	0.0194 (4)	0.0265 (4)
Mo2	0.0216 (4)	0.0246 (4)	0.0324 (4)	0.0105 (3)	−0.0006 (3)	0.0024 (3)
Mo3	0.0343 (4)	0.0240 (4)	0.0177 (3)	−0.0009 (3)	−0.0001 (3)	0.0100 (3)
Mo4	0.0420 (4)	0.0319 (4)	0.0445 (5)	0.0213 (3)	0.0178 (4)	0.0275 (4)
Mo5	0.0186 (3)	0.0259 (4)	0.0233 (4)	0.0030 (3)	−0.0013 (3)	0.0012 (3)
Mo6	0.0231 (4)	0.0267 (4)	0.0315 (4)	0.0119 (3)	−0.0024 (3)	0.0057 (3)
Mo7	0.0224 (4)	0.0394 (4)	0.0444 (5)	0.0028 (3)	0.0076 (3)	0.0277 (4)
Mo8	0.0262 (4)	0.0404 (4)	0.0415 (5)	0.0063 (3)	0.0129 (3)	0.0263 (4)
Mo9	0.0255 (4)	0.0198 (3)	0.0254 (4)	0.0084 (3)	−0.0012 (3)	0.0016 (3)
O1	0.024 (3)	0.026 (3)	0.020 (3)	0.007 (2)	0.001 (2)	0.007 (2)
O2	0.026 (3)	0.017 (2)	0.011 (2)	0.006 (2)	0.000 (2)	0.0031 (19)
O3	0.030 (3)	0.022 (3)	0.022 (3)	0.008 (2)	−0.003 (2)	0.007 (2)
O4	0.028 (3)	0.027 (3)	0.019 (3)	0.009 (2)	0.002 (2)	0.007 (2)
O5	0.027 (3)	0.023 (3)	0.022 (3)	0.009 (2)	0.003 (2)	0.004 (2)
O6	0.027 (3)	0.025 (3)	0.022 (3)	0.009 (2)	0.002 (2)	0.006 (2)
O7	0.032 (3)	0.020 (3)	0.019 (3)	0.006 (2)	0.006 (2)	0.007 (2)
O8	0.030 (3)	0.045 (4)	0.036 (3)	0.019 (3)	−0.008 (3)	−0.009 (3)
O9	0.026 (3)	0.038 (3)	0.034 (3)	0.014 (3)	−0.001 (2)	0.001 (3)
O10	0.028 (3)	0.033 (3)	0.025 (3)	0.011 (2)	0.005 (2)	0.007 (2)
O11	0.029 (3)	0.025 (3)	0.020 (3)	0.003 (2)	0.002 (2)	0.008 (2)
O12	0.029 (3)	0.026 (3)	0.019 (3)	0.004 (2)	−0.003 (2)	0.009 (2)
O13	0.032 (3)	0.049 (4)	0.021 (3)	−0.007 (3)	0.001 (2)	0.011 (3)
O14	0.050 (4)	0.020 (3)	0.031 (3)	0.009 (3)	−0.003 (3)	0.014 (2)
O15	0.066 (4)	0.024 (3)	0.022 (3)	0.023 (3)	0.004 (3)	0.010 (2)
O16	0.036 (3)	0.038 (3)	0.027 (3)	−0.006 (3)	0.011 (3)	0.009 (3)
O17	0.027 (3)	0.041 (3)	0.033 (3)	0.009 (3)	0.001 (3)	0.006 (3)
O18	0.036 (3)	0.022 (3)	0.028 (3)	0.007 (2)	0.006 (2)	0.002 (2)
O19	0.038 (3)	0.034 (3)	0.019 (3)	0.000 (3)	−0.003 (2)	0.010 (2)
O20	0.055 (4)	0.018 (3)	0.037 (3)	0.007 (3)	−0.013 (3)	0.009 (3)
O21	0.028 (3)	0.048 (4)	0.024 (3)	−0.008 (3)	0.006 (2)	0.007 (3)
O22	0.028 (3)	0.055 (4)	0.040 (4)	0.024 (3)	−0.008 (3)	0.004 (3)
O23	0.043 (4)	0.050 (4)	0.051 (4)	0.014 (3)	0.009 (3)	0.020 (3)
O24	0.043 (4)	0.046 (4)	0.051 (4)	0.015 (3)	0.001 (3)	0.011 (3)
O25	0.082 (6)	0.169 (9)	0.035 (4)	0.104 (6)	0.023 (4)	0.042 (5)
O26	0.051 (4)	0.048 (4)	0.053 (4)	0.015 (3)	0.004 (3)	0.018 (3)
O27	0.096 (6)	0.021 (3)	0.085 (6)	−0.003 (4)	−0.060 (5)	0.020 (4)
O28	0.050 (4)	0.066 (5)	0.068 (5)	0.031 (4)	−0.023 (4)	−0.029 (4)
O29	0.034 (4)	0.110 (7)	0.032 (4)	−0.020 (4)	−0.001 (3)	0.016 (4)
O30	0.168 (9)	0.038 (4)	0.032 (4)	0.039 (5)	0.019 (5)	0.012 (3)
O31	0.068 (5)	0.033 (4)	0.080 (5)	−0.018 (3)	0.050 (4)	−0.007 (4)
C1	0.050 (6)	0.035 (5)	0.052 (6)	0.013 (5)	0.017 (5)	0.010 (5)
C2	0.045 (6)	0.052 (6)	0.047 (6)	0.014 (5)	−0.001 (5)	0.028 (5)
C3	0.049 (6)	0.028 (4)	0.034 (5)	0.011 (4)	0.010 (4)	0.008 (4)
C4	0.036 (5)	0.063 (7)	0.036 (5)	0.005 (5)	0.001 (4)	0.025 (5)
C5	0.076 (8)	0.051 (7)	0.080 (9)	0.032 (6)	0.045 (7)	0.037 (6)
C6	0.073 (8)	0.062 (7)	0.029 (5)	0.028 (6)	−0.008 (5)	−0.005 (5)
C7	0.076 (8)	0.067 (8)	0.039 (6)	0.046 (7)	−0.018 (6)	−0.004 (5)
C8	0.029 (5)	0.026 (4)	0.041 (5)	−0.003 (4)	−0.006 (4)	0.007 (4)
C9	0.037 (5)	0.022 (4)	0.035 (5)	−0.005 (4)	0.004 (4)	0.014 (4)
C10	0.050 (6)	0.049 (6)	0.056 (6)	0.013 (5)	0.016 (5)	0.035 (5)
C11	0.040 (5)	0.042 (5)	0.020 (4)	0.021 (4)	0.000 (4)	0.006 (4)
C12	0.042 (6)	0.085 (8)	0.070 (7)	0.030 (6)	0.011 (5)	0.050 (7)
C13	0.063 (7)	0.073 (8)	0.078 (8)	0.047 (6)	0.030 (6)	0.054 (7)
C14	0.031 (5)	0.077 (7)	0.033 (5)	0.028 (5)	0.009 (4)	0.016 (5)
C15	0.042 (5)	0.071 (7)	0.032 (5)	0.034 (5)	0.013 (4)	0.023 (5)
N1	0.038 (5)	0.031 (4)	0.081 (7)	0.011 (4)	−0.004 (5)	0.002 (4)
N2	0.046 (5)	0.031 (5)	0.080 (7)	0.012 (4)	−0.001 (5)	0.002 (5)
N3	0.046 (5)	0.049 (5)	0.054 (5)	0.017 (4)	0.029 (4)	0.016 (4)

### Geometric parameters (Å, °)

**Table:**

As1—O26	1.678 (7)	Mo8—O21	1.671 (5)
As1—O23	1.682 (7)	Mo8—O27	1.837 (6)
As1—O2	1.696 (4)	Mo8—O5	1.885 (5)
As1—O24	1.698 (7)	Mo8—O28	1.915 (7)
Mo1—O20	1.664 (5)	Mo8—O13^i^	2.007 (5)
Mo1—O25	1.829 (6)	Mo8—O26	2.347 (7)
Mo1—O29	1.889 (7)	Mo9—O18	1.701 (5)
Mo1—O15	1.913 (5)	Mo9—O10	1.798 (5)
Mo1—O1	1.981 (5)	Mo9—O1	1.917 (5)
Mo1—O23	2.329 (7)	Mo9—O7	1.960 (5)
Mo2—O9	1.673 (5)	Mo9—O11	2.052 (5)
Mo2—O8	1.768 (5)	Mo9—O2	2.371 (5)
Mo2—O27	1.854 (6)	O8—Mo6^i^	2.066 (5)
Mo2—O29	1.950 (7)	O13—Mo8^i^	2.007 (5)
Mo2—O10	2.185 (5)	O15—Mo4^i^	1.941 (5)
Mo2—O23	2.327 (6)	C1—N1	1.345 (13)
Mo3—O19	1.687 (5)	C1—C3	1.350 (13)
Mo3—O12	1.882 (5)	C1—H1B	0.9300
Mo3—O3	1.934 (5)	C2—N1	1.313 (13)
Mo3—O7	1.936 (5)	C2—C4	1.385 (14)
Mo3—O5	1.971 (5)	C2—H2A	0.9300
Mo3—O2	2.365 (5)	C3—C9	1.393 (12)
Mo3—Mo5	3.3719 (10)	C3—H3A	0.9300
Mo3—Mo9	3.3976 (10)	C4—C9	1.387 (12)
Mo4—O14	1.705 (5)	C4—H4A	0.9300
Mo4—O31	1.820 (6)	C5—N2	1.307 (14)
Mo4—O28	1.905 (8)	C5—C10	1.344 (15)
Mo4—O15^i^	1.941 (5)	C5—H5A	0.9300
Mo4—O3	1.951 (5)	C6—C8	1.375 (13)
Mo4—O26	2.290 (7)	C6—C7	1.408 (15)
Mo4—Mo8	3.4372 (11)	C6—H6A	0.9300
Mo5—O17	1.695 (5)	C7—N2	1.293 (14)
Mo5—O11	1.823 (5)	C7—H7A	0.9300
Mo5—O6	1.847 (5)	C8—C10	1.393 (13)
Mo5—O12	1.991 (5)	C8—C9	1.465 (13)
Mo5—O4	2.055 (5)	C10—H10A	0.9300
Mo5—O2	2.383 (5)	C11—C15	1.371 (12)
Mo5—Mo9	3.4021 (10)	C11—C12	1.377 (13)
Mo6—O22	1.655 (5)	C11—C11^ii^	1.501 (17)
Mo6—O4	1.805 (5)	C12—C13	1.363 (14)
Mo6—O31	1.901 (6)	C12—H12A	0.9300
Mo6—O30	1.902 (7)	C13—N3	1.308 (12)
Mo6—O8^i^	2.066 (5)	C13—H13A	0.9300
Mo6—O24	2.358 (7)	C14—C15	1.335 (13)
Mo6—Mo7	3.4319 (11)	C14—N3	1.391 (12)
Mo7—O16	1.659 (5)	C14—H14A	0.9300
Mo7—O13	1.858 (5)	C15—H15A	0.9300
Mo7—O25	1.866 (6)	N1—H1A	0.8600
Mo7—O30	1.923 (8)	N2—H2B	0.8600
Mo7—O6	2.051 (5)	N3—H3B	0.8600
Mo7—O24	2.294 (7)		
			
O26—As1—O23	110.6 (3)	O25—Mo7—O24	90.9 (3)
O26—As1—O2	108.6 (3)	O30—Mo7—O24	67.5 (3)
O23—As1—O2	108.6 (3)	O6—Mo7—O24	81.1 (2)
O26—As1—O24	109.5 (3)	O16—Mo7—Mo6	122.1 (2)
O23—As1—O24	110.1 (3)	O13—Mo7—Mo6	100.1 (2)
O2—As1—O24	109.4 (3)	O25—Mo7—Mo6	131.3 (3)
O20—Mo1—O25	103.2 (4)	O30—Mo7—Mo6	26.0 (3)
O20—Mo1—O29	99.3 (4)	O6—Mo7—Mo6	73.78 (14)
O25—Mo1—O29	157.2 (4)	O24—Mo7—Mo6	43.18 (16)
O20—Mo1—O15	98.0 (2)	O21—Mo8—O27	105.2 (4)
O25—Mo1—O15	89.7 (3)	O21—Mo8—O5	98.7 (2)
O29—Mo1—O15	91.0 (3)	O27—Mo8—O5	91.5 (2)
O20—Mo1—O1	94.4 (2)	O21—Mo8—O28	98.9 (4)
O25—Mo1—O1	89.3 (2)	O27—Mo8—O28	155.5 (4)
O29—Mo1—O1	85.1 (2)	O5—Mo8—O28	88.8 (2)
O15—Mo1—O1	167.5 (2)	O21—Mo8—O13^i^	95.4 (2)
O20—Mo1—O23	165.5 (3)	O27—Mo8—O13^i^	86.3 (3)
O25—Mo1—O23	91.1 (4)	O5—Mo8—O13^i^	165.8 (2)
O29—Mo1—O23	66.2 (3)	O28—Mo8—O13^i^	87.4 (3)
O15—Mo1—O23	84.1 (2)	O21—Mo8—O26	164.4 (3)
O1—Mo1—O23	83.4 (2)	O27—Mo8—O26	90.2 (4)
O9—Mo2—O8	102.4 (3)	O5—Mo8—O26	83.3 (2)
O9—Mo2—O27	103.9 (4)	O28—Mo8—O26	65.5 (3)
O8—Mo2—O27	97.3 (3)	O13^i^—Mo8—O26	82.7 (2)
O9—Mo2—O29	97.4 (3)	O21—Mo8—Mo4	123.5 (2)
O8—Mo2—O29	96.4 (3)	O27—Mo8—Mo4	131.2 (3)
O27—Mo2—O29	151.5 (3)	O5—Mo8—Mo4	78.21 (15)
O9—Mo2—O10	87.7 (2)	O28—Mo8—Mo4	25.8 (3)
O8—Mo2—O10	169.5 (2)	O13^i^—Mo8—Mo4	92.66 (19)
O27—Mo2—O10	82.8 (2)	O26—Mo8—Mo4	41.54 (16)
O29—Mo2—O10	79.3 (2)	O18—Mo9—O10	105.1 (2)
O9—Mo2—O23	160.7 (3)	O18—Mo9—O1	100.4 (2)
O8—Mo2—O23	88.9 (2)	O10—Mo9—O1	90.3 (2)
O27—Mo2—O23	89.9 (3)	O18—Mo9—O7	101.4 (2)
O29—Mo2—O23	65.5 (3)	O10—Mo9—O7	92.7 (2)
O10—Mo2—O23	80.6 (2)	O1—Mo9—O7	156.3 (2)
O19—Mo3—O12	100.9 (2)	O18—Mo9—O11	95.5 (2)
O19—Mo3—O3	102.6 (2)	O10—Mo9—O11	159.4 (2)
O12—Mo3—O3	90.6 (2)	O1—Mo9—O11	85.6 (2)
O19—Mo3—O7	98.9 (2)	O7—Mo9—O11	83.3 (2)
O12—Mo3—O7	91.5 (2)	O18—Mo9—O2	165.9 (2)
O3—Mo3—O7	157.6 (2)	O10—Mo9—O2	88.3 (2)
O19—Mo3—O5	101.8 (2)	O1—Mo9—O2	83.47 (19)
O12—Mo3—O5	157.3 (2)	O7—Mo9—O2	73.19 (18)
O3—Mo3—O5	83.1 (2)	O11—Mo9—O2	71.19 (18)
O7—Mo3—O5	86.5 (2)	O18—Mo9—Mo3	129.50 (19)
O19—Mo3—O2	171.3 (2)	O10—Mo9—Mo3	91.20 (17)
O12—Mo3—O2	75.12 (19)	O1—Mo9—Mo3	127.42 (15)
O3—Mo3—O2	85.29 (19)	O7—Mo9—Mo3	29.11 (14)
O7—Mo3—O2	73.75 (18)	O11—Mo9—Mo3	75.51 (14)
O5—Mo3—O2	82.58 (19)	O2—Mo9—Mo3	44.08 (11)
O19—Mo3—Mo5	130.11 (18)	O18—Mo9—Mo5	122.26 (18)
O12—Mo3—Mo5	30.39 (15)	O10—Mo9—Mo5	132.63 (17)
O3—Mo3—Mo5	91.13 (16)	O1—Mo9—Mo5	82.16 (15)
O7—Mo3—Mo5	79.64 (15)	O7—Mo9—Mo5	78.56 (15)
O5—Mo3—Mo5	127.55 (15)	O11—Mo9—Mo5	26.80 (14)
O2—Mo3—Mo5	44.97 (11)	O2—Mo9—Mo5	44.45 (11)
O19—Mo3—Mo9	128.2 (2)	Mo3—Mo9—Mo5	59.46 (2)
O12—Mo3—Mo9	83.42 (16)	Mo9—O1—Mo1	144.0 (3)
O3—Mo3—Mo9	129.06 (15)	As1—O2—Mo3	125.2 (2)
O7—Mo3—Mo9	29.52 (14)	As1—O2—Mo9	123.6 (2)
O5—Mo3—Mo9	83.73 (15)	Mo3—O2—Mo9	91.69 (16)
O2—Mo3—Mo9	44.24 (11)	As1—O2—Mo5	124.5 (2)
Mo5—Mo3—Mo9	60.34 (2)	Mo3—O2—Mo5	90.51 (16)
O14—Mo4—O31	101.5 (4)	Mo9—O2—Mo5	91.38 (16)
O14—Mo4—O28	98.9 (3)	Mo3—O3—Mo4	149.4 (3)
O31—Mo4—O28	159.5 (4)	Mo6—O4—Mo5	146.6 (3)
O14—Mo4—O15^i^	96.6 (2)	Mo8—O5—Mo3	148.9 (3)
O31—Mo4—O15^i^	89.3 (3)	Mo5—O6—Mo7	151.5 (3)
O28—Mo4—O15^i^	89.5 (3)	Mo3—O7—Mo9	121.4 (2)
O14—Mo4—O3	96.2 (2)	Mo2—O8—Mo6^i^	168.5 (3)
O31—Mo4—O3	89.8 (3)	Mo9—O10—Mo2	145.1 (3)
O28—Mo4—O3	86.7 (2)	Mo5—O11—Mo9	122.7 (3)
O15^i^—Mo4—O3	167.1 (2)	Mo3—O12—Mo5	121.0 (3)
O14—Mo4—O26	165.8 (3)	Mo7—O13—Mo8^i^	167.9 (3)
O31—Mo4—O26	92.6 (3)	Mo1—O15—Mo4^i^	168.2 (3)
O28—Mo4—O26	66.9 (3)	As1—O23—Mo2	125.0 (3)
O15^i^—Mo4—O26	84.4 (2)	As1—O23—Mo1	121.6 (3)
O3—Mo4—O26	82.7 (2)	Mo2—O23—Mo1	97.1 (2)
O14—Mo4—Mo8	123.2 (2)	As1—O24—Mo7	124.1 (3)
O31—Mo4—Mo8	134.0 (3)	As1—O24—Mo6	120.7 (3)
O28—Mo4—Mo8	25.9 (3)	Mo7—O24—Mo6	95.1 (2)
O15^i^—Mo4—Mo8	95.15 (18)	Mo1—O25—Mo7	156.5 (6)
O3—Mo4—Mo8	76.28 (15)	As1—O26—Mo4	124.0 (3)
O26—Mo4—Mo8	42.80 (17)	As1—O26—Mo8	121.0 (3)
O17—Mo5—O11	103.4 (2)	Mo4—O26—Mo8	95.7 (2)
O17—Mo5—O6	103.9 (3)	Mo8—O27—Mo2	158.2 (6)
O11—Mo5—O6	97.6 (2)	Mo4—O28—Mo8	128.3 (5)
O17—Mo5—O12	97.6 (2)	Mo1—O29—Mo2	130.8 (5)
O11—Mo5—O12	88.9 (2)	Mo6—O30—Mo7	127.6 (5)
O6—Mo5—O12	155.4 (2)	Mo4—O31—Mo6	151.9 (5)
O17—Mo5—O4	99.2 (2)	N1—C1—C3	118.5 (9)
O11—Mo5—O4	156.5 (2)	N1—C1—H1B	120.7
O6—Mo5—O4	83.1 (2)	C3—C1—H1B	120.7
O12—Mo5—O4	81.8 (2)	N1—C2—C4	119.1 (9)
O17—Mo5—O2	170.2 (2)	N1—C2—H2A	120.4
O11—Mo5—O2	74.61 (19)	C4—C2—H2A	120.4
O6—Mo5—O2	85.92 (19)	C1—C3—C9	121.1 (9)
O12—Mo5—O2	72.86 (18)	C1—C3—H3A	119.4
O4—Mo5—O2	82.01 (18)	C9—C3—H3A	119.4
O17—Mo5—Mo3	125.8 (2)	C2—C4—C9	119.7 (9)
O11—Mo5—Mo3	78.62 (16)	C2—C4—H4A	120.2
O6—Mo5—Mo3	129.90 (16)	C9—C4—H4A	120.2
O12—Mo5—Mo3	28.57 (14)	N2—C5—C10	120.1 (10)
O4—Mo5—Mo3	82.99 (14)	N2—C5—H5A	119.9
O2—Mo5—Mo3	44.53 (11)	C10—C5—H5A	119.9
O17—Mo5—Mo9	133.62 (19)	C8—C6—C7	120.3 (10)
O11—Mo5—Mo9	30.51 (15)	C8—C6—H6A	119.8
O6—Mo5—Mo9	91.66 (16)	C7—C6—H6A	119.8
O12—Mo5—Mo9	81.85 (15)	N2—C7—C6	118.5 (10)
O4—Mo5—Mo9	126.19 (14)	N2—C7—H7A	120.7
O2—Mo5—Mo9	44.17 (11)	C6—C7—H7A	120.7
Mo3—Mo5—Mo9	60.21 (2)	C6—C8—C10	116.3 (9)
O22—Mo6—O4	102.1 (3)	C6—C8—C9	122.2 (9)
O22—Mo6—O31	102.1 (4)	C10—C8—C9	121.5 (8)
O4—Mo6—O31	93.7 (3)	C4—C9—C3	117.7 (9)
O22—Mo6—O30	100.3 (4)	C4—C9—C8	120.6 (8)
O4—Mo6—O30	91.8 (3)	C3—C9—C8	121.6 (8)
O31—Mo6—O30	155.2 (4)	C5—C10—C8	121.0 (10)
O22—Mo6—O8^i^	93.8 (3)	C5—C10—H10A	119.5
O4—Mo6—O8^i^	164.1 (2)	C8—C10—H10A	119.5
O31—Mo6—O8^i^	82.5 (3)	C15—C11—C12	116.0 (9)
O30—Mo6—O8^i^	85.6 (3)	C15—C11—C11^ii^	121.6 (9)
O22—Mo6—O24	165.5 (3)	C12—C11—C11^ii^	122.3 (9)
O4—Mo6—O24	84.9 (2)	C13—C12—C11	122.0 (9)
O31—Mo6—O24	90.0 (3)	C13—C12—H12A	119.0
O30—Mo6—O24	66.4 (3)	C11—C12—H12A	119.0
O8^i^—Mo6—O24	79.7 (2)	N3—C13—C12	119.4 (9)
O22—Mo6—Mo7	126.1 (2)	N3—C13—H13A	120.3
O4—Mo6—Mo7	81.29 (16)	C12—C13—H13A	120.3
O31—Mo6—Mo7	131.7 (3)	C15—C14—N3	118.0 (8)
O30—Mo6—Mo7	26.4 (3)	C15—C14—H14A	121.0
O8^i^—Mo6—Mo7	89.71 (18)	N3—C14—H14A	121.0
O24—Mo6—Mo7	41.75 (16)	C14—C15—C11	122.9 (9)
O16—Mo7—O13	99.1 (3)	C14—C15—H15A	118.5
O16—Mo7—O25	102.2 (4)	C11—C15—H15A	118.5
O13—Mo7—O25	90.9 (3)	C2—N1—C1	123.8 (9)
O16—Mo7—O30	98.5 (4)	C2—N1—H1A	118.1
O13—Mo7—O30	94.9 (3)	C1—N1—H1A	118.1
O25—Mo7—O30	157.3 (4)	C7—N2—C5	123.8 (10)
O16—Mo7—O6	93.0 (2)	C7—N2—H2B	118.1
O13—Mo7—O6	167.9 (2)	C5—N2—H2B	118.1
O25—Mo7—O6	85.7 (2)	C13—N3—C14	121.5 (8)
O30—Mo7—O6	84.0 (2)	C13—N3—H3B	119.3
O16—Mo7—O24	165.2 (3)	C14—N3—H3B	119.3
O13—Mo7—O24	87.4 (2)		
			
O19—Mo3—Mo5—O17	−7.7 (4)	O5—Mo3—O3—Mo4	−26.0 (6)
O12—Mo3—Mo5—O17	10.7 (4)	O2—Mo3—O3—Mo4	57.0 (6)
O3—Mo3—Mo5—O17	99.9 (3)	Mo5—Mo3—O3—Mo4	101.7 (6)
O7—Mo3—Mo5—O17	−100.6 (3)	Mo9—Mo3—O3—Mo4	50.1 (7)
O5—Mo3—Mo5—O17	−178.0 (3)	O14—Mo4—O3—Mo3	143.2 (6)
O2—Mo3—Mo5—O17	−177.9 (3)	O31—Mo4—O3—Mo3	−115.2 (6)
Mo9—Mo3—Mo5—O17	−124.6 (2)	O28—Mo4—O3—Mo3	44.6 (6)
O19—Mo3—Mo5—O11	90.7 (3)	O15^i^—Mo4—O3—Mo3	−28.9 (15)
O12—Mo3—Mo5—O11	109.1 (3)	O26—Mo4—O3—Mo3	−22.5 (6)
O3—Mo3—Mo5—O11	−161.7 (2)	Mo8—Mo4—O3—Mo3	20.6 (5)
O7—Mo3—Mo5—O11	−2.2 (2)	O22—Mo6—O4—Mo5	−136.8 (5)
O5—Mo3—Mo5—O11	−79.6 (2)	O31—Mo6—O4—Mo5	120.0 (6)
O2—Mo3—Mo5—O11	−79.6 (2)	O30—Mo6—O4—Mo5	−35.9 (6)
Mo9—Mo3—Mo5—O11	−26.17 (16)	O8^i^—Mo6—O4—Mo5	44.4 (12)
O19—Mo3—Mo5—O6	−179.0 (3)	O24—Mo6—O4—Mo5	30.3 (5)
O12—Mo3—Mo5—O6	−160.6 (4)	Mo7—Mo6—O4—Mo5	−11.7 (5)
O3—Mo3—Mo5—O6	−71.4 (3)	O17—Mo5—O4—Mo6	121.5 (5)
O7—Mo3—Mo5—O6	88.1 (3)	O11—Mo5—O4—Mo6	−74.6 (8)
O5—Mo3—Mo5—O6	10.8 (3)	O6—Mo5—O4—Mo6	18.5 (5)
O2—Mo3—Mo5—O6	10.8 (3)	O12—Mo5—O4—Mo6	−142.1 (5)
Mo9—Mo3—Mo5—O6	64.2 (2)	O2—Mo5—O4—Mo6	−68.3 (5)
O19—Mo3—Mo5—O12	−18.4 (4)	Mo3—Mo5—O4—Mo6	−113.3 (5)
O3—Mo3—Mo5—O12	89.2 (3)	Mo9—Mo5—O4—Mo6	−68.5 (6)
O7—Mo3—Mo5—O12	−111.3 (3)	O21—Mo8—O5—Mo3	−139.1 (6)
O5—Mo3—Mo5—O12	171.4 (4)	O27—Mo8—O5—Mo3	115.2 (6)
O2—Mo3—Mo5—O12	171.4 (4)	O28—Mo8—O5—Mo3	−40.3 (6)
Mo9—Mo3—Mo5—O12	−135.3 (3)	O13^i^—Mo8—O5—Mo3	34.4 (13)
O19—Mo3—Mo5—O4	−104.1 (3)	O26—Mo8—O5—Mo3	25.2 (6)
O12—Mo3—Mo5—O4	−85.7 (3)	Mo4—Mo8—O5—Mo3	−16.6 (5)
O3—Mo3—Mo5—O4	3.53 (18)	O19—Mo3—O5—Mo8	124.6 (6)
O7—Mo3—Mo5—O4	163.0 (2)	O12—Mo3—O5—Mo8	−51.7 (9)
O5—Mo3—Mo5—O4	85.7 (2)	O3—Mo3—O5—Mo8	23.1 (6)
O2—Mo3—Mo5—O4	85.7 (2)	O7—Mo3—O5—Mo8	−137.1 (6)
Mo9—Mo3—Mo5—O4	139.10 (14)	O2—Mo3—O5—Mo8	−63.1 (6)
O19—Mo3—Mo5—O2	170.2 (3)	Mo5—Mo3—O5—Mo8	−63.1 (6)
O12—Mo3—Mo5—O2	−171.4 (4)	Mo9—Mo3—O5—Mo8	−107.6 (6)
O3—Mo3—Mo5—O2	−82.2 (2)	O17—Mo5—O6—Mo7	−125.5 (6)
O7—Mo3—Mo5—O2	77.3 (2)	O11—Mo5—O6—Mo7	128.7 (6)
O5—Mo3—Mo5—O2	0.0 (2)	O12—Mo5—O6—Mo7	24.7 (10)
Mo9—Mo3—Mo5—O2	53.38 (16)	O4—Mo5—O6—Mo7	−27.6 (6)
O19—Mo3—Mo5—Mo9	116.8 (3)	O2—Mo5—O6—Mo7	54.8 (6)
O12—Mo3—Mo5—Mo9	135.3 (3)	Mo3—Mo5—O6—Mo7	47.2 (7)
O3—Mo3—Mo5—Mo9	−135.57 (14)	Mo9—Mo5—O6—Mo7	98.6 (6)
O7—Mo3—Mo5—Mo9	23.94 (15)	O16—Mo7—O6—Mo5	146.0 (6)
O5—Mo3—Mo5—Mo9	−53.40 (18)	O13—Mo7—O6—Mo5	−37.8 (16)
O2—Mo3—Mo5—Mo9	−53.38 (16)	O25—Mo7—O6—Mo5	−111.9 (7)
O22—Mo6—Mo7—O16	13.4 (3)	O30—Mo7—O6—Mo5	47.8 (7)
O4—Mo6—Mo7—O16	−85.3 (3)	O24—Mo7—O6—Mo5	−20.3 (6)
O31—Mo6—Mo7—O16	−172.5 (4)	Mo6—Mo7—O6—Mo5	23.4 (6)
O30—Mo6—Mo7—O16	27.2 (5)	O19—Mo3—O7—Mo9	−175.1 (3)
O8^i^—Mo6—Mo7—O16	107.8 (3)	O12—Mo3—O7—Mo9	−73.8 (3)
O24—Mo6—Mo7—O16	−177.4 (3)	O3—Mo3—O7—Mo9	21.2 (7)
O22—Mo6—Mo7—O13	−94.2 (3)	O5—Mo3—O7—Mo9	83.5 (3)
O4—Mo6—Mo7—O13	167.1 (2)	O2—Mo3—O7—Mo9	0.2 (3)
O31—Mo6—Mo7—O13	79.9 (3)	Mo5—Mo3—O7—Mo9	−45.7 (2)
O30—Mo6—Mo7—O13	−80.4 (5)	O18—Mo9—O7—Mo3	166.4 (3)
O8^i^—Mo6—Mo7—O13	0.2 (2)	O10—Mo9—O7—Mo3	−87.6 (3)
O24—Mo6—Mo7—O13	75.0 (3)	O1—Mo9—O7—Mo3	9.4 (7)
O22—Mo6—Mo7—O25	165.4 (4)	O11—Mo9—O7—Mo3	72.1 (3)
O4—Mo6—Mo7—O25	66.8 (4)	O2—Mo9—O7—Mo3	−0.2 (3)
O31—Mo6—Mo7—O25	−20.4 (4)	Mo5—Mo9—O7—Mo3	45.4 (2)
O30—Mo6—Mo7—O25	179.3 (6)	O9—Mo2—O8—Mo6^i^	168 (2)
O8^i^—Mo6—Mo7—O25	−100.1 (4)	O27—Mo2—O8—Mo6^i^	62 (2)
O24—Mo6—Mo7—O25	−25.3 (4)	O29—Mo2—O8—Mo6^i^	−93 (2)
O22—Mo6—Mo7—O30	−13.8 (5)	O10—Mo2—O8—Mo6^i^	−28 (3)
O4—Mo6—Mo7—O30	−112.5 (5)	O23—Mo2—O8—Mo6^i^	−28 (2)
O31—Mo6—Mo7—O30	160.3 (6)	O18—Mo9—O10—Mo2	−127.2 (5)
O8^i^—Mo6—Mo7—O30	80.6 (5)	O1—Mo9—O10—Mo2	−26.3 (5)
O24—Mo6—Mo7—O30	155.4 (5)	O7—Mo9—O10—Mo2	130.2 (5)
O22—Mo6—Mo7—O6	96.5 (3)	O11—Mo9—O10—Mo2	52.2 (9)
O4—Mo6—Mo7—O6	−2.1 (2)	O2—Mo9—O10—Mo2	57.2 (5)
O31—Mo6—Mo7—O6	−89.3 (3)	Mo3—Mo9—O10—Mo2	101.2 (5)
O30—Mo6—Mo7—O6	110.4 (5)	Mo5—Mo9—O10—Mo2	53.3 (6)
O8^i^—Mo6—Mo7—O6	−169.0 (2)	O9—Mo2—O10—Mo9	143.0 (6)
O24—Mo6—Mo7—O6	−94.2 (3)	O8—Mo2—O10—Mo9	−21.6 (18)
O22—Mo6—Mo7—O24	−169.3 (3)	O27—Mo2—O10—Mo9	−112.7 (6)
O4—Mo6—Mo7—O24	92.1 (3)	O29—Mo2—O10—Mo9	45.0 (6)
O31—Mo6—Mo7—O24	4.9 (3)	O23—Mo2—O10—Mo9	−21.6 (5)
O30—Mo6—Mo7—O24	−155.4 (5)	O17—Mo5—O11—Mo9	173.3 (3)
O8^i^—Mo6—Mo7—O24	−74.8 (3)	O6—Mo5—O11—Mo9	−80.4 (3)
O14—Mo4—Mo8—O21	3.3 (3)	O12—Mo5—O11—Mo9	75.8 (3)
O31—Mo4—Mo8—O21	167.8 (4)	O4—Mo5—O11—Mo9	9.7 (7)
O28—Mo4—Mo8—O21	−19.9 (5)	O2—Mo5—O11—Mo9	3.3 (2)
O15^i^—Mo4—Mo8—O21	−98.1 (3)	Mo3—Mo5—O11—Mo9	48.9 (2)
O3—Mo4—Mo8—O21	91.8 (3)	O18—Mo9—O11—Mo5	−178.7 (3)
O26—Mo4—Mo8—O21	−174.1 (3)	O10—Mo9—O11—Mo5	1.9 (8)
O14—Mo4—Mo8—O27	−171.1 (3)	O1—Mo9—O11—Mo5	81.2 (3)
O31—Mo4—Mo8—O27	−6.5 (4)	O7—Mo9—O11—Mo5	−77.8 (3)
O28—Mo4—Mo8—O27	165.8 (5)	O2—Mo9—O11—Mo5	−3.4 (3)
O15^i^—Mo4—Mo8—O27	87.6 (3)	Mo3—Mo9—O11—Mo5	−49.3 (3)
O3—Mo4—Mo8—O27	−82.6 (3)	O19—Mo3—O12—Mo5	165.8 (3)
O26—Mo4—Mo8—O27	11.5 (4)	O3—Mo3—O12—Mo5	−91.3 (3)
O14—Mo4—Mo8—O5	−89.5 (3)	O7—Mo3—O12—Mo5	66.5 (3)
O31—Mo4—Mo8—O5	75.1 (4)	O5—Mo3—O12—Mo5	−18.0 (7)
O28—Mo4—Mo8—O5	−112.6 (5)	O2—Mo3—O12—Mo5	−6.3 (3)
O15^i^—Mo4—Mo8—O5	169.2 (2)	Mo9—Mo3—O12—Mo5	38.0 (3)
O3—Mo4—Mo8—O5	−1.0 (2)	O17—Mo5—O12—Mo3	−171.3 (3)
O26—Mo4—Mo8—O5	93.1 (3)	O11—Mo5—O12—Mo3	−67.9 (3)
O14—Mo4—Mo8—O28	23.1 (5)	O6—Mo5—O12—Mo3	37.9 (7)
O31—Mo4—Mo8—O28	−172.3 (6)	O4—Mo5—O12—Mo3	90.4 (3)
O15^i^—Mo4—Mo8—O28	−78.2 (5)	O2—Mo5—O12—Mo3	6.3 (3)
O3—Mo4—Mo8—O28	111.6 (5)	Mo9—Mo5—O12—Mo3	−38.1 (3)
O26—Mo4—Mo8—O28	−154.3 (5)	O16—Mo7—O13—Mo8^i^	−179.8 (19)
O14—Mo4—Mo8—O13^i^	101.6 (3)	O25—Mo7—O13—Mo8^i^	77.6 (19)
O31—Mo4—Mo8—O13^i^	−93.9 (4)	O30—Mo7—O13—Mo8^i^	−80.4 (19)
O28—Mo4—Mo8—O13^i^	78.4 (5)	O6—Mo7—O13—Mo8^i^	4(3)
O15^i^—Mo4—Mo8—O13^i^	0.2 (2)	O24—Mo7—O13—Mo8^i^	−13.3 (19)
O3—Mo4—Mo8—O13^i^	−169.9 (2)	Mo6—Mo7—O13—Mo8^i^	−54.7 (19)
O26—Mo4—Mo8—O13^i^	−75.8 (3)	O20—Mo1—O15—Mo4^i^	−176.8 (18)
O14—Mo4—Mo8—O26	177.4 (3)	O25—Mo1—O15—Mo4^i^	−73.5 (18)
O31—Mo4—Mo8—O26	−18.0 (4)	O29—Mo1—O15—Mo4^i^	83.7 (18)
O28—Mo4—Mo8—O26	154.3 (5)	O1—Mo1—O15—Mo4^i^	12 (3)
O15^i^—Mo4—Mo8—O26	76.1 (3)	O23—Mo1—O15—Mo4^i^	17.7 (18)
O3—Mo4—Mo8—O26	−94.1 (3)	O26—As1—O23—Mo2	−53.2 (5)
O19—Mo3—Mo9—O18	−11.2 (3)	O2—As1—O23—Mo2	65.9 (4)
O12—Mo3—Mo9—O18	87.5 (3)	O24—As1—O23—Mo2	−174.3 (3)
O3—Mo3—Mo9—O18	172.8 (3)	O26—As1—O23—Mo1	179.3 (3)
O7—Mo3—Mo9—O18	−17.4 (4)	O2—As1—O23—Mo1	−61.6 (4)
O5—Mo3—Mo9—O18	−111.4 (3)	O24—As1—O23—Mo1	58.2 (4)
O2—Mo3—Mo9—O18	162.9 (3)	O9—Mo2—O23—As1	−112.0 (8)
Mo5—Mo3—Mo9—O18	108.5 (2)	O8—Mo2—O23—As1	121.6 (4)
O19—Mo3—Mo9—O10	99.6 (3)	O27—Mo2—O23—As1	24.4 (4)
O12—Mo3—Mo9—O10	−161.7 (2)	O29—Mo2—O23—As1	−140.8 (5)
O3—Mo3—Mo9—O10	−76.4 (3)	O10—Mo2—O23—As1	−58.4 (4)
O7—Mo3—Mo9—O10	93.4 (3)	O9—Mo2—O23—Mo1	25.0 (9)
O5—Mo3—Mo9—O10	−0.5 (2)	O8—Mo2—O23—Mo1	−101.3 (3)
O2—Mo3—Mo9—O10	−86.3 (2)	O27—Mo2—O23—Mo1	161.4 (3)
Mo5—Mo3—Mo9—O10	−140.73 (16)	O29—Mo2—O23—Mo1	−3.8 (2)
O19—Mo3—Mo9—O1	−169.1 (3)	O10—Mo2—O23—Mo1	78.7 (2)
O12—Mo3—Mo9—O1	−70.4 (2)	O20—Mo1—O23—As1	137.5 (9)
O3—Mo3—Mo9—O1	15.0 (3)	O25—Mo1—O23—As1	−33.8 (4)
O7—Mo3—Mo9—O1	−175.3 (4)	O29—Mo1—O23—As1	142.9 (5)
O5—Mo3—Mo9—O1	90.8 (2)	O15—Mo1—O23—As1	−123.4 (4)
O2—Mo3—Mo9—O1	5.0 (2)	O1—Mo1—O23—As1	55.4 (4)
Mo5—Mo3—Mo9—O1	−49.38 (18)	O20—Mo1—O23—Mo2	−1.5 (11)
O19—Mo3—Mo9—O7	6.2 (4)	O25—Mo1—O23—Mo2	−172.9 (3)
O12—Mo3—Mo9—O7	104.9 (3)	O29—Mo1—O23—Mo2	3.9 (2)
O3—Mo3—Mo9—O7	−169.8 (4)	O15—Mo1—O23—Mo2	97.6 (3)
O5—Mo3—Mo9—O7	−93.9 (3)	O1—Mo1—O23—Mo2	−83.7 (2)
O2—Mo3—Mo9—O7	−179.7 (3)	O26—As1—O24—Mo7	−177.6 (3)
Mo5—Mo3—Mo9—O7	125.9 (3)	O23—As1—O24—Mo7	−55.8 (5)
O19—Mo3—Mo9—O11	−96.3 (3)	O2—As1—O24—Mo7	63.5 (4)
O12—Mo3—Mo9—O11	2.4 (2)	O26—As1—O24—Mo6	60.7 (4)
O3—Mo3—Mo9—O11	87.7 (2)	O23—As1—O24—Mo6	−177.5 (3)
O7—Mo3—Mo9—O11	−102.5 (3)	O2—As1—O24—Mo6	−58.2 (4)
O5—Mo3—Mo9—O11	163.6 (2)	O16—Mo7—O24—As1	−124.0 (9)
O2—Mo3—Mo9—O11	77.8 (2)	O13—Mo7—O24—As1	119.4 (4)
Mo5—Mo3—Mo9—O11	23.37 (14)	O25—Mo7—O24—As1	28.5 (4)
O19—Mo3—Mo9—O2	−174.1 (3)	O30—Mo7—O24—As1	−144.1 (5)
O12—Mo3—Mo9—O2	−75.4 (2)	O6—Mo7—O24—As1	−57.0 (4)
O3—Mo3—Mo9—O2	10.0 (2)	Mo6—Mo7—O24—As1	−132.7 (5)
O7—Mo3—Mo9—O2	179.7 (3)	O16—Mo7—O24—Mo6	8.7 (11)
O5—Mo3—Mo9—O2	85.8 (2)	O13—Mo7—O24—Mo6	−107.8 (3)
Mo5—Mo3—Mo9—O2	−54.39 (16)	O25—Mo7—O24—Mo6	161.3 (3)
O19—Mo3—Mo9—Mo5	−119.7 (2)	O30—Mo7—O24—Mo6	−11.4 (2)
O12—Mo3—Mo9—Mo5	−21.01 (15)	O6—Mo7—O24—Mo6	75.8 (2)
O3—Mo3—Mo9—Mo5	64.3 (2)	O22—Mo6—O24—As1	171.9 (9)
O7—Mo3—Mo9—Mo5	−125.9 (3)	O4—Mo6—O24—As1	52.3 (4)
O5—Mo3—Mo9—Mo5	140.18 (15)	O31—Mo6—O24—As1	−41.4 (4)
O2—Mo3—Mo9—Mo5	54.39 (16)	O30—Mo6—O24—As1	146.6 (4)
O17—Mo5—Mo9—O18	−7.4 (4)	O8^i^—Mo6—O24—As1	−123.8 (4)
O11—Mo5—Mo9—O18	1.5 (4)	Mo7—Mo6—O24—As1	135.0 (5)
O6—Mo5—Mo9—O18	103.6 (3)	O22—Mo6—O24—Mo7	36.9 (11)
O12—Mo5—Mo9—O18	−100.2 (3)	O4—Mo6—O24—Mo7	−82.6 (2)
O4—Mo5—Mo9—O18	−173.7 (3)	O31—Mo6—O24—Mo7	−176.4 (3)
O2—Mo5—Mo9—O18	−173.9 (3)	O30—Mo6—O24—Mo7	11.6 (2)
Mo3—Mo5—Mo9—O18	−120.1 (2)	O8^i^—Mo6—O24—Mo7	101.3 (3)
O17—Mo5—Mo9—O10	172.0 (4)	O20—Mo1—O25—Mo7	−167.3 (11)
O11—Mo5—Mo9—O10	−179.1 (4)	O29—Mo1—O25—Mo7	2.7 (17)
O6—Mo5—Mo9—O10	−77.0 (3)	O15—Mo1—O25—Mo7	94.6 (11)
O12—Mo5—Mo9—O10	79.2 (3)	O1—Mo1—O25—Mo7	−72.9 (11)
O4—Mo5—Mo9—O10	5.7 (3)	O23—Mo1—O25—Mo7	10.5 (11)
O2—Mo5—Mo9—O10	5.5 (3)	O16—Mo7—O25—Mo1	164.8 (11)
Mo3—Mo5—Mo9—O10	59.3 (2)	O13—Mo7—O25—Mo1	−95.7 (11)
O17—Mo5—Mo9—O1	−104.8 (3)	O30—Mo7—O25—Mo1	9.6 (17)
O11—Mo5—Mo9—O1	−95.9 (3)	O6—Mo7—O25—Mo1	72.7 (11)
O6—Mo5—Mo9—O1	6.2 (2)	O24—Mo7—O25—Mo1	−8.3 (11)
O12—Mo5—Mo9—O1	162.4 (2)	Mo6—Mo7—O25—Mo1	8.8 (13)
O4—Mo5—Mo9—O1	88.9 (2)	O23—As1—O26—Mo4	−177.8 (3)
O2—Mo5—Mo9—O1	88.6 (2)	O2—As1—O26—Mo4	63.1 (4)
Mo3—Mo5—Mo9—O1	142.52 (15)	O24—As1—O26—Mo4	−56.3 (5)
O17—Mo5—Mo9—O7	88.9 (3)	O23—As1—O26—Mo8	59.2 (5)
O11—Mo5—Mo9—O7	97.9 (3)	O2—As1—O26—Mo8	−59.9 (4)
O6—Mo5—Mo9—O7	−160.0 (2)	O24—As1—O26—Mo8	−179.3 (3)
O12—Mo5—Mo9—O7	−3.8 (2)	O14—Mo4—O26—As1	−142.6 (8)
O4—Mo5—Mo9—O7	−77.4 (2)	O31—Mo4—O26—As1	33.4 (4)
O2—Mo5—Mo9—O7	−77.6 (2)	O28—Mo4—O26—As1	−145.6 (5)
Mo3—Mo5—Mo9—O7	−23.72 (14)	O15^i^—Mo4—O26—As1	122.5 (4)
O17—Mo5—Mo9—O11	−9.0 (4)	O3—Mo4—O26—As1	−56.1 (4)
O6—Mo5—Mo9—O11	102.1 (3)	Mo8—Mo4—O26—As1	−133.7 (5)
O12—Mo5—Mo9—O11	−101.7 (3)	O14—Mo4—O26—Mo8	−8.9 (11)
O4—Mo5—Mo9—O11	−175.2 (3)	O31—Mo4—O26—Mo8	167.1 (3)
O2—Mo5—Mo9—O11	−175.5 (3)	O28—Mo4—O26—Mo8	−11.9 (2)
Mo3—Mo5—Mo9—O11	−121.6 (3)	O15^i^—Mo4—O26—Mo8	−103.8 (3)
O17—Mo5—Mo9—O2	166.5 (3)	O3—Mo4—O26—Mo8	77.7 (2)
O11—Mo5—Mo9—O2	175.5 (3)	O21—Mo8—O26—As1	154.1 (7)
O6—Mo5—Mo9—O2	−82.4 (2)	O27—Mo8—O26—As1	−35.7 (4)
O12—Mo5—Mo9—O2	73.8 (2)	O5—Mo8—O26—As1	55.8 (4)
O4—Mo5—Mo9—O2	0.2 (2)	O28—Mo8—O26—As1	147.6 (5)
Mo3—Mo5—Mo9—O2	53.87 (16)	O13^i^—Mo8—O26—As1	−121.9 (4)
O17—Mo5—Mo9—Mo3	112.6 (3)	Mo4—Mo8—O26—As1	135.7 (5)
O11—Mo5—Mo9—Mo3	121.6 (3)	O21—Mo8—O26—Mo4	18.4 (10)
O6—Mo5—Mo9—Mo3	−136.29 (15)	O27—Mo8—O26—Mo4	−171.3 (3)
O12—Mo5—Mo9—Mo3	19.88 (14)	O5—Mo8—O26—Mo4	−79.8 (2)
O4—Mo5—Mo9—Mo3	−53.63 (17)	O28—Mo8—O26—Mo4	12.0 (2)
O2—Mo5—Mo9—Mo3	−53.87 (16)	O13^i^—Mo8—O26—Mo4	102.4 (3)
O18—Mo9—O1—Mo1	126.5 (5)	O21—Mo8—O27—Mo2	−173.5 (11)
O10—Mo9—O1—Mo1	21.1 (5)	O5—Mo8—O27—Mo2	−74.0 (11)
O7—Mo9—O1—Mo1	−76.4 (7)	O28—Mo8—O27—Mo2	16.5 (15)
O11—Mo9—O1—Mo1	−138.7 (5)	O13^i^—Mo8—O27—Mo2	91.9 (11)
O2—Mo9—O1—Mo1	−67.2 (5)	O26—Mo8—O27—Mo2	9.2 (11)
Mo3—Mo9—O1—Mo1	−70.7 (5)	Mo4—Mo8—O27—Mo2	1.6 (13)
Mo5—Mo9—O1—Mo1	−112.0 (5)	O9—Mo2—O27—Mo8	162.0 (11)
O20—Mo1—O1—Mo9	−136.9 (5)	O8—Mo2—O27—Mo8	−93.3 (11)
O25—Mo1—O1—Mo9	119.9 (6)	O29—Mo2—O27—Mo8	24.8 (16)
O29—Mo1—O1—Mo9	−37.9 (5)	O10—Mo2—O27—Mo8	76.2 (11)
O15—Mo1—O1—Mo9	34.4 (14)	O23—Mo2—O27—Mo8	−4.4 (11)
O23—Mo1—O1—Mo9	28.7 (5)	O14—Mo4—O28—Mo8	−160.6 (4)
O26—As1—O2—Mo3	−2.9 (4)	O31—Mo4—O28—Mo8	16.0 (11)
O23—As1—O2—Mo3	−123.3 (3)	O15^i^—Mo4—O28—Mo8	102.8 (5)
O24—As1—O2—Mo3	116.5 (3)	O3—Mo4—O28—Mo8	−64.8 (4)
O26—As1—O2—Mo9	117.8 (3)	O26—Mo4—O28—Mo8	18.7 (4)
O23—As1—O2—Mo9	−2.5 (4)	O21—Mo8—O28—Mo4	163.3 (4)
O24—As1—O2—Mo9	−122.7 (3)	O27—Mo8—O28—Mo4	−26.5 (9)
O26—As1—O2—Mo5	−122.8 (3)	O5—Mo8—O28—Mo4	64.7 (5)
O23—As1—O2—Mo5	116.9 (3)	O13^i^—Mo8—O28—Mo4	−101.6 (5)
O24—As1—O2—Mo5	−3.3 (4)	O26—Mo8—O28—Mo4	−18.4 (4)
O19—Mo3—O2—As1	166.6 (14)	O20—Mo1—O29—Mo2	172.6 (4)
O12—Mo3—O2—As1	−129.8 (3)	O25—Mo1—O29—Mo2	2.4 (11)
O3—Mo3—O2—As1	−38.0 (3)	O15—Mo1—O29—Mo2	−89.2 (4)
O7—Mo3—O2—As1	134.1 (3)	O1—Mo1—O29—Mo2	78.9 (4)
O5—Mo3—O2—As1	45.6 (3)	O23—Mo1—O29—Mo2	−6.1 (4)
Mo5—Mo3—O2—As1	−134.3 (4)	O9—Mo2—O29—Mo1	−164.7 (4)
Mo9—Mo3—O2—As1	134.3 (4)	O8—Mo2—O29—Mo1	91.9 (4)
O19—Mo3—O2—Mo9	32.4 (16)	O27—Mo2—O29—Mo1	−26.3 (9)
O12—Mo3—O2—Mo9	95.9 (2)	O10—Mo2—O29—Mo1	−78.4 (4)
O3—Mo3—O2—Mo9	−172.26 (19)	O23—Mo2—O29—Mo1	6.1 (4)
O7—Mo3—O2—Mo9	−0.14 (18)	O22—Mo6—O30—Mo7	168.7 (4)
O5—Mo3—O2—Mo9	−88.62 (18)	O4—Mo6—O30—Mo7	66.1 (5)
Mo5—Mo3—O2—Mo9	91.40 (16)	O31—Mo6—O30—Mo7	−36.9 (9)
O19—Mo3—O2—Mo5	−59.0 (16)	O8^i^—Mo6—O30—Mo7	−98.3 (5)
O12—Mo3—O2—Mo5	4.50 (18)	O24—Mo6—O30—Mo7	−17.6 (4)
O3—Mo3—O2—Mo5	96.34 (19)	O16—Mo7—O30—Mo6	−157.0 (4)
O7—Mo3—O2—Mo5	−91.54 (19)	O13—Mo7—O30—Mo6	103.0 (5)
O5—Mo3—O2—Mo5	179.98 (19)	O25—Mo7—O30—Mo6	−1.4 (11)
Mo9—Mo3—O2—Mo5	−91.40 (16)	O6—Mo7—O30—Mo6	−64.8 (4)
O18—Mo9—O2—As1	155.6 (8)	O24—Mo7—O30—Mo6	17.9 (4)
O10—Mo9—O2—As1	−41.9 (3)	O14—Mo4—O31—Mo6	149.3 (10)
O1—Mo9—O2—As1	48.6 (3)	O28—Mo4—O31—Mo6	−27.2 (17)
O7—Mo9—O2—As1	−135.2 (3)	O15^i^—Mo4—O31—Mo6	−114.1 (10)
O11—Mo9—O2—As1	136.2 (3)	O3—Mo4—O31—Mo6	52.9 (10)
Mo3—Mo9—O2—As1	−135.4 (4)	O26—Mo4—O31—Mo6	−29.8 (11)
Mo5—Mo9—O2—As1	134.1 (4)	Mo8—Mo4—O31—Mo6	−17.6 (12)
O18—Mo9—O2—Mo3	−69.0 (9)	O22—Mo6—O31—Mo4	−154.8 (10)
O10—Mo9—O2—Mo3	93.5 (2)	O4—Mo6—O31—Mo4	−51.6 (11)
O1—Mo9—O2—Mo3	−176.00 (19)	O30—Mo6—O31—Mo4	50.9 (14)
O7—Mo9—O2—Mo3	0.14 (18)	O8^i^—Mo6—O31—Mo4	112.9 (11)
O11—Mo9—O2—Mo3	−88.39 (19)	O24—Mo6—O31—Mo4	33.3 (11)
Mo5—Mo9—O2—Mo3	−90.55 (16)	Mo7—Mo6—O31—Mo4	30.0 (12)
O18—Mo9—O2—Mo5	21.5 (9)	N1—C1—C3—C9	0.4 (13)
O10—Mo9—O2—Mo5	−176.0 (2)	N1—C2—C4—C9	3.0 (14)
O1—Mo9—O2—Mo5	−85.45 (18)	C8—C6—C7—N2	−0.9 (16)
O7—Mo9—O2—Mo5	90.69 (19)	C7—C6—C8—C10	0.2 (15)
O11—Mo9—O2—Mo5	2.16 (16)	C7—C6—C8—C9	−178.8 (9)
Mo3—Mo9—O2—Mo5	90.55 (16)	C2—C4—C9—C3	−1.1 (13)
O17—Mo5—O2—As1	144.7 (12)	C2—C4—C9—C8	−178.4 (8)
O11—Mo5—O2—As1	−135.8 (3)	C1—C3—C9—C4	−0.5 (13)
O6—Mo5—O2—As1	−36.8 (3)	C1—C3—C9—C8	176.7 (8)
O12—Mo5—O2—As1	130.6 (3)	C6—C8—C9—C4	−171.0 (9)
O4—Mo5—O2—As1	46.7 (3)	C10—C8—C9—C4	10.0 (12)
Mo3—Mo5—O2—As1	134.9 (4)	C6—C8—C9—C3	11.8 (13)
Mo9—Mo5—O2—As1	−133.4 (4)	C10—C8—C9—C3	−167.1 (8)
O17—Mo5—O2—Mo3	9.9 (14)	N2—C5—C10—C8	−0.6 (16)
O11—Mo5—O2—Mo3	89.3 (2)	C6—C8—C10—C5	0.5 (14)
O6—Mo5—O2—Mo3	−171.70 (19)	C9—C8—C10—C5	179.5 (9)
O12—Mo5—O2—Mo3	−4.31 (18)	C15—C11—C12—C13	1.8 (16)
O4—Mo5—O2—Mo3	−88.11 (18)	C11^ii^—C11—C12—C13	−180.0 (11)
Mo9—Mo5—O2—Mo3	91.70 (16)	C11—C12—C13—N3	−1.7 (18)
O17—Mo5—O2—Mo9	−81.8 (13)	N3—C14—C15—C11	−3.0 (14)
O11—Mo5—O2—Mo9	−2.39 (18)	C12—C11—C15—C14	0.6 (14)
O6—Mo5—O2—Mo9	96.60 (19)	C11^ii^—C11—C15—C14	−177.6 (10)
O12—Mo5—O2—Mo9	−96.0 (2)	C4—C2—N1—C1	−3.3 (15)
O4—Mo5—O2—Mo9	−179.81 (18)	C3—C1—N1—C2	1.6 (14)
Mo3—Mo5—O2—Mo9	−91.70 (16)	C6—C7—N2—C5	0.9 (17)
O19—Mo3—O3—Mo4	−126.7 (6)	C10—C5—N2—C7	−0.2 (17)
O12—Mo3—O3—Mo4	132.1 (6)	C12—C13—N3—C14	−0.9 (16)
O7—Mo3—O3—Mo4	36.8 (10)	C15—C14—N3—C13	3.1 (14)

Symmetry codes: (i) −*x*+2, −*y*+1, −*z*+1; (ii) −*x*+1, −*y*+1, −*z*.

### Hydrogen-bond geometry (Å, °)

**Table:**

*D*—H···*A*	*D*—H	H···*A*	*D*···*A*	*D*—H···*A*
N1—H1A···O14	0.86	1.99	2.851 (10)	174
N2—H2B···O29^iii^	0.86	2.32	2.805 (12)	116
N3—H3B···O10^iv^	0.86	2.56	3.291 (9)	143
N3—H3B···O7^iv^	0.86	2.60	3.336 (10)	145

Symmetry codes: (iii) *x*−1, *y*+1, *z*; (iv) *x*−1, *y*, *z*.
